# CALR accelerates the growth of liver cancer cells by enhancing telomere activity via ARAF

**DOI:** 10.1016/j.gendis.2025.101715

**Published:** 2025-06-14

**Authors:** Sijie Xie, Xiaoxue Jiang, Xinlei Liu, Shuting Song, Liyan Wang, Shujie Li, Dongdong Lu

**Affiliations:** Shanghai Putuo People's Hospital, School of Life Science and Technology, Tongji University, Shanghai 200092, China

Calreticulin (CALR) is a pleiotropic and highly conserved molecule and is recognized as an unfolded protein response effector protein. Moreover, CALR is an endoplasmic reticulum protein involved in a range of cellular processes. CALR can be translocated from the endoplasmic reticulum to the cell surface through co-localization with protein disulfide isomerase family A member 3 (PDIA3).^1^ Furthermore, CALR mutations affected the spindle assembly checkpoint, leading to erroneous mitosis.^2^ In particular, the loss-of-function CALR mutations not only impair cellular homeostasis but also compromise both natural and therapy-driven immune surveillance, thereby promoting tumorigenesis.^3^ Also, CALR frameshift mutations, a primary cause of myeloproliferative neoplasms, lead to rogue interactions with the thrombopoietin receptor (TpoR).^4^ Type I CALR mutations, but not type II, activate the inositol-requiring enzyme 1α (IRE1α)/X-box binding protein 1 (XBP1) pathway of the unfolded protein response, driving the development of myeloproliferative neoplasms.^5^ In this study, we demonstrate that CALR accelerates the growth of liver cancer cells by enhancing telomere activity dependent on ARAF (A-Raf proto-oncogene, serine/threonine kinase). Therefore, these results provide a basis for research on liver cancer prevention and treatment.

To address the effect of CALR on liver cancer cells, we cloned the CALR into the lentiviral vector pLVX-ZsGreen-Puro (pLVX-CALR) and prepared rLV-CALR lentivirus. Next, liver cancer cell Huh 7 cells were infected using rLV and rLV-CALR (Fig. S1A). CALR was overexpressed in the rLV-CALR group compared with the rLV group (Fig. 1A, B). The proliferation ability (Fig. 1C), cellular colony formation ability (28.53% ± 5.13% *vs.* 69.04% ± 8.97%; *p* = 0.00026) (Fig. 1D; Fig. S1B), and tumor formation ability (0.295 ± 0.054 *vs.* 0.885 ± 0.09 g; *p* = 0.0000014) (Fig. 1E–G; Fig. S1C–E) were significantly increased in the rLV-CALR group compared with the rLV group. Furthermore, CALR was decreased in the rLV-shRNA CALR (1) group and rLV-shRNA CALR (2) group compared with the rLV-shRNA (Fig. S2A, B). The proliferation ability, the cellular colony formation ability, and tumor formation ability were significantly decreased in the rLV-shRNA CALR (1) group and rLV-shRNA CALR (2) group compared with the rLV-shRNA group (Fig. S2C–J). Collectively, these results suggest that CALR accelerates the growth ability of liver cancer cells to grow *in vivo* and *in vitro.*

**Figure 1:**
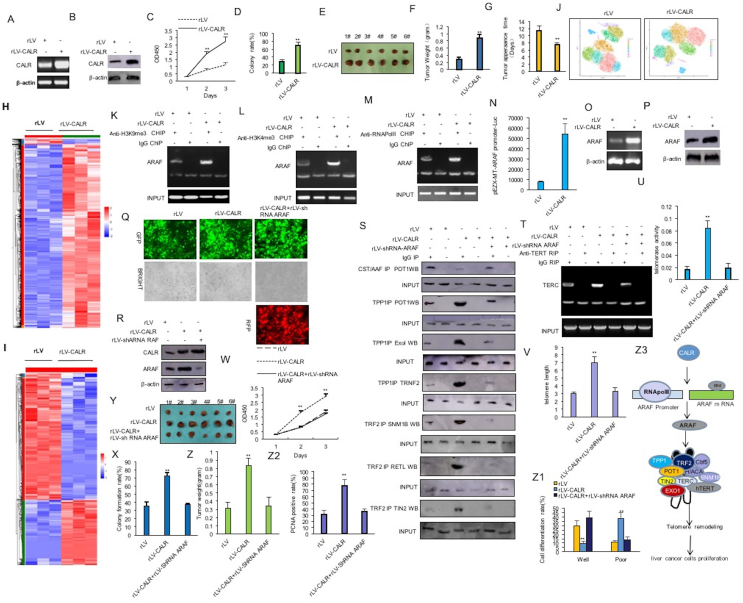
CALR accelerates the growth of liver cancer cells by enhancing telomere activity via ARAF. **(A)** CALR was detected by reverse transcription PCR. β-actin was used as the internal reference gene. **(B)** CALR was detected by western blotting with anti-CALR. β-actin was used as the internal reference gene. **(C)** CCK8 method was used to determine the cell proliferation ability. The values of each group were expressed as mean ± standard deviation (*n* = 6); ∗∗*p* < 0.01 and ∗*p* < 0.05. **(D)** The colony-forming ability of cells was measured. The values of each group were expressed as mean ± standard deviation (*n* = 6); ∗∗*p* < 0.01 and ∗*p* < 0.05. **(E)** The xenograft tumor was dissected. **(F)** Comparison of tumor size (g). **(G)** The appearance time of the tumor (days). The values of each group were expressed as mean ± standard deviation (*n* = 6); ∗∗*p* < 0.01 and ∗*p* < 0.05. **(H)** RNA sequencing analysis: Heatmap analysis (cluster) of gene expression in the two groups. **(I)** Protein sequencing analysis: The differential protein cluster heatmap. The vertical is the clustering of samples, and the horizontal is the clustering of proteins. **(J)** Single-cell RNA-sequencing analysis: The dimensionality reduction clustering t-SNE diagram. **(K**–**M)** Chromatin immunoprecipitation (ChIP) analysis was performed by anti-H3K97me3, anti-H3K4me3, and anti-RNApolII. The PCR amplification was carried out using primers designed according to the DNA of the ARAF promoter region. IgG ChIP was used as the negative control. **(N)** The assay of pEZX-MT-ARAF-promoter-Luc activity. **(O)** The transcriptional ability of ARAF was detected by reverse-transcription PCR. β-actin was used as the internal reference gene. **(P)** The translational ability of ARAF was detected by western blotting. β-actin was used as the internal reference gene. **(Q)** Huh 7 cells were infected with rLV, rLV-CALR, and rLV-CALR + rLV-shRNA ARAF, and the images were taken under a fluorescence microscope. **(R)** CALR and ARAF were detected by western blotting. β-actin is used as the internal reference. **(S)** Protein co-immunoprecipitation (Co-IP) analysis was performed. IgG Co-IP was used as the negative control. **(T)** RNA immunoprecipitation (RIP) analysis was performed by anti-TERT. The reverse-transcription PCR amplification was carried out using primers designed according to TERC cDNA. IgG RIP was used as the negative control. **(U)** The assay of telomerase activity. ∗∗*p* < 0.01 and ∗*p* < 0.05. **(V)** The assay of telomere length. ∗∗*p* < 0.01 and ∗*p* < 0.05. **(W)** The proliferation ability was determined by CCK8. ∗∗*p* < 0.01 and ∗*p* < 0.05. **(X)** The colony formation ability of the cells was measured. The values of each group were expressed as mean ± standard error of the mean (*n* = 6); ∗∗*p* < 0.01 and ∗*p* < 0.05. **(Y)** The xenograft tumor was dissected. **(Z)** Comparison of tumor size (g). The values of each group were expressed as mean ± standard deviation (*n* = 6); ∗∗*p* < 0.01 and ∗*p* < 0.05. **(Z1)** Hematoxylin and eosin staining analysis. The values of each group were expressed as mean ± standard deviation (*n* = 6); ∗∗*p* < 0.01 and ∗*p* < 0.05. **(Z2)** Anti-PCNA staining. The values of each group were expressed as mean ± standard deviation (*n* = 6); ∗∗*p* < 0.01 and ∗*p* < 0.05. **(Z3)** The schematic diagram of the molecular mechanism by which CALR accelerates the growth of liver cancer cells by enhancing telomere activity via ARAF. CALR, calreticulin; ARAF, A-Raf proto-oncogene, serine/threonine kinase; TERT, telomerase reverse transcriptase; PCNA, proliferating cell nuclear antigen.

Furthermore, the results of chromatin immunoprecipitation followed by sequencing (Fig. S3A–G) and assay for transposase-accessible chromatin using sequencing (Fig. S3H) showed that CALR affected epigenetic regulation of genes, *e.g.*, ARAF. Moreover, RNA sequencing (Fig. 1H) and protein sequencing (Fig. 1I) indicate that CALR could affect gene expression (*e.g.*, ARAF) by altering transcriptome and proteome in human liver cancer cells. Importantly, single-cell RNA sequencing showed that CALR affected the heterogeneity of liver cancer and its microenvironment network, involving ARAF (Fig. 1J; Fig. S4A–F, Fig.S5A–I). In particular, the binding ability of H3K9me3, H3K4me3, and RNAPolII to the promoter region of ARAF was significantly increased in the rLV-CALR group compared with the rLV group (Fig. 1K–M; Fig. S6A–D). The ATAF promoter luciferase activity was significantly increased in the rLV-CALR group compared with the rLV group (7447.19 ± 1044.46 *vs.* 54396.27 ± 9891.46; *p* = 0.0058) (Fig. 1N). The binding ability of methyltransferase 3 (METTL3) to ARAF mRNA (Fig. S6E), methylation modification ability of ARAF mRNA (Fig. S6F), ARAF 3′-UTR luciferase activity (Fig. S6G), and expression ability of ARAF (Fig. 1O, P) were significantly increased in the rLV-CALR group compared with the rLV group. Furthermore, the interaction between ARAF and cyclin D1 (CCND1), N-Ras, Y-box binding protein 1 (YB-1), X-ray repair cross complementing 5 (XRCC5), or c-Myc was significantly increased, and the interaction between ARAF and Zic family member 1 (ZIC1), retinoblastoma 1 (RB1), growth arrest and DNA damage-inducible 45 (GADD45), chromobox protein 3 (CBX3), or P21 (WAF1/CIP1) was decreased in the rLV-CALR group compared with the rLV group (Fig. S6H).

As shown in Figure 1Q and R, CALR was significantly increased in the rLV-CALR group and the rLV-CALR + rLV-shRNA-ARAF group compared with the rLV group, and AFAR was significantly increased in the rLV-CALR group and decreased in the rLV-CALR + rLV-shRNA-ARAF group compared with the rLV group. Although the binding ability of METTL3 to K-Ras mRNA and telomerase reverse transcriptase (TERT) mRNA, the methylation modification ability of K-Ras mRNA and TERT mRNA, and the expression ability of K-Ras, TERT, CCND1, c-Myc, glycogen synthase kinase 3 Beta (GSK3β), and proliferating cell nuclear antigen (PCNA) were significantly increased, and the binding ability of METTL3 to phosphatase and tensin homolog (PTEN) mRNA and RB1 mRNA, the methylation modification ability of PTEN mRNA and RB1 mRNA, and the expression ability of P57, RB1, PTEN, cullin 5 (CUL5), and P73 were significantly decreased in the rLV-CALR group compared with the rLV group, these were not significantly changed in the rLV-CALR + rLV-shRNA-ARAF group versus the rLV group (Fig. S7A–D). Although the interaction between tripeptidyl peptidase 1 (TPP1) and protection of telomeres 1 (POT1) or exonuclease 1 (ExoI), telomeric repeat binding factor 2 (TRNF2) and sensitivity to nitrogen mustard 1 (SNM1B), regulator of telomere elongation helicase (RETL), or TERF1 interacting nuclear factor 2 (TIN2), TERT and centromere/microtubule binding protein 5 (Cbf5), TERT and telomerase Cajal body protein 1 (TCAB1), TERT and Reptin, and TERT and Pontin were significantly increased and the interaction between CTC1-STN1-TEN1 (CST)/alpha accessory factor (AAF) and POT1 was significantly decreased in the rLV-CALR group compared with the rLV group, these were not significantly changed in the rLV-CALR + rLV-shRNA-ARAF group versus the rLV group (Fig. 1S; Fig. S8A–G, 9A). Although the interaction between TERT and telomerase RNA component (TERC) (Fig. 1T) and the interaction between Cbf5 and H/ACA (Fig. S9B), telomerase activity (0.0167 ± 0.0046 *vs.* 0.0849 ± 0.011; *p* = 0.000006) (Fig. 1U), or telomere length (3.01 ± 0.147 *vs.* 6.98 ± 0.743; *p* = 0.00395) (Fig. 1V) were significantly increased and the interaction between TERT and telomeric repeat-containing RNA (TERRA) (Fig. S9C) was significantly decreased in the rLV-CALR group compared with the rLV group, these were not significantly changed in the rLV-CALR + rLV-shRNA-ARAF group versus the rLV group. Taken together, these observations suggest that CALR alters gene expression and the telomerase activity dependent on ARAF in liver cancer. Although the proliferation ability (24 h: *p* = 0.00148; 48 h: *p* = 0.0011) (Fig. 1W), colony formation ability (35.603% ± 4.89% *vs.* 72.81% ± 13.98%; *p* = 0.0059) (Fig. 1X; Fig. S10A), and tumorigenesis ability of transplanted tumors [tumor weight: 0.221 ± 0.036 g *vs*. 0.675 ± 0.105 g (*p* = 0.00002; Fig. 1Y, Z); tumor appearance time: 11.83 ± 2.14 days *vs.* 7.33 ± 1.21 days (*p* = 0.003; Fig. S9B); well differentiated cells: 30.12% ± 5.34% *vs.* 9.87% ± 2.34% (*p* = 0.0019; Fig. 1Z1); poorly differentiated cells:11.23% ± 2.09% *vs.* 37.89% ± 8.1% (*p* = 0.0033; Fig. 1Z1); PCNA positive rate: 31.11% ± 7.03% *vs.* 77.42% ± 10.16% (*p* = 0.00008; Fig. 1Z2; Fig. S10C)] were significantly increased in the rLV-CALR group compared with the rLV group, these were not significantly changed in the rLV-CALR + rLV-shRNA-ARAF group versus the rLV group. Taken together, these observations suggest that CALR enhances the carcinogenic function via ARAF in liver cancer.

In conclusion, we clearly demonstrate that CALR accelerates the growth of liver cancer cells by enhancing ARAF expression and telomere function (Fig. 1Z3). This first discovery provides a basis for the prevention and treatment of human liver cancer.

## CRediT authorship contribution statement

**Sijie Xie:** Investigation. **Xiaoxue Jiang:** Investigation. **Xinlei Liu:** Investigation. **Shuting Song:** Investigation. **Liyan Wang:** Investigation. **Shujie Li:** Investigation. **Dongdong Lu:** Writing – review & editing, Writing – original draft, Visualization, Validation, Supervision, Software, Resources, Project administration, Methodology, Investigation, Funding acquisition, Formal analysis, Data curation, Conceptualization.

## Ethics declaration

All methods were carried out in accordance with the approved guidelines. All experimental protocols were approved by a Tongji University institutional committee. Informed consent was obtained from all subjects. The study was reviewed and approved by the China National Institutional Animal Care and Use Committee.

## Funding

This study was supported by a grant from the 10.13039/501100001809National Natural Science Foundation of China (No. 82073130).

## Conflict of interests

The authors declared no competing interests.
