# Supplementary material for: CALR accelerates the growth of liver cancer cells by enhancing telomere activity via ARAF
Source: Genes Dis. 2025 Jun 14;13(1):101715. doi: 10.1016/j.gendis.2025.101715 (PMC12624556; doi:10.1016/j.gendis.2025.101715)
Supplement: Multimedia component 1 [file mmc1.docx]

**Supplemental Data**

**Materials and Methods**

**Cell Lines, Lentivirus** Human liver cancer cell line (Huh7) was maintained in Dulbecco’s modified Eagle medium (Gibco BRL Life Technologies) in a humidified atmosphere of 5% CO_2_ incubator at 37ºC. rLV, rLV-CALR were purchased from Wu Han viraltherapy Technologies Co. Ltd.

**Cell infection and transfection.** Cells were infected with Lentivirus and transfected with DNA plasmids using transfast transfection reagent lipofectamine^R^ 2000 (Invitrogen) according to manufacturer's instructions.

**RT-PCR** Total RNA was purified using Trizol (Invitrogen) according to manufacturer's instructions. cDNA was prepared by SuperScript First-Strand Synthesis System (Invitrogen). PCR analysis was performed according to our previous reports. β-actin was used as an internal control.

**Western blotting** Total proteins were separated on a 10% sodium dodecyl sulfate-polyacrylamide gel electrophoresis (SDS-PAGE) and transferred onto a nitrocellulose membrane. The blots were incubated with antibody at 4°C overnight. Following three washes, membranes were then incubated with secondary antibody at 4°C overnight. Signals were visualized by ECL system.

**Co-immunoprecipitation(IP)** Cells were lysed in 1 ml of the whole-cell extract buffer A(50mM pH7.6 Tris-HCl, 150mMNaCl, 1%NP40, 0.1mMEDTA,1.0mM DTT,0.2mMPMSF, 0.1mM Pepstatine,0.1mM Leupeptine,0.1mM Aproine). Five-hundred-microliter cell lysates was used in immunoprecipitation with antibody. Western blot was performed with the another related antibody indicated in Western blotting.

**Chromatin immunoprecipitation (ChIP) assay** Cells were cross-linked with 1% (v/v) formaldehyde (Sigma) for 10 min at room temperature and stopped with 125 mm glycine for 5 min. Crossed-linked cells were washed with phosphate-buffered saline, resuspended in lysis buffer, and sonicated for 8 min. Chromatin extracts were pre-cleared with Protein-A/G-Sepharose beads, and immunoprecipitated with specific antibody on Protein-A/G-Sepharose beads. After washing, elution and de-cross-linking, the ChIP DNA was detected by PCR).

**Determination of telomerase activity and telomere length** Telomerase activity in cells was detected using the quantitative telomerase activity assay (TRAP) method (Roche, Basel, CH, Switzerland). Cellular DNA was extracted and telomere length was analyzed by quantitative PCR amplification using the Absolute Human Telomere Length Quantification qPCR Assay Kit (ScienCell) and telomere-specific primers (Forward 5’-TTTAGGGTTAGGGTTAGGGT-3’, Reverse 5’-CCTAACCCTAACCCTAACCC-3’). The DNA amplification quantity of β-actin was used as an internal reference.

### Cell proliferation ability Assay The cell proliferation abilty was measured using CCK8 kit and the operation according to the manufacturer instruction(Beyotime Biotechnology).

**Colony-Formation Efficiency Assay** 10^3^ cells were plated on a six-wells and the 2 ml DMEM containing 10%FBS was added into each well of the three replicate. Then cells were incubated at 37°C in humidified incubator for 7 days. Cell colonies were stained with 1 ml of 0. 05% Crystal Violet for more than 1 hour and the colonies were counted.

**Xenograft transplantation *in vivo*** Four-weeks male athymic Balb/C mice per group were injected with liver cancer cells Huh7 at the armpit area subcutaneously. The mice were observed over 4 weeks, and then sacrificed to recover the tumors. The use of mice for this work was reviewed and approved by the institutional animal care and use committee in accordance with China national institutes of health guidelines.

**Chip-Seq** ChIP sequencing analysis was performed according to according to the manufacturer operation manual (Novogene Co., Ltd., Beijing Nuohe Zhiyuan Technology Co., Ltd.)

**RNA sequencing analysis** RNA sequencing analysis was performed according to according to the manufacturer operation manual (Shanghai Majorbio Bio-pharm Technology Co.,Ltd).

**Mass spectrometric analysis** Mass spectrometric analysis of enzyme hydrolyzed peptides of protein without label free was performed according to according to the manufacturer operation manual (Shanghai Majorbio Bio-pharm Technology Co.,Ltd)

**Immunoprecipitation protein mass spectrometry** Immunoprecipitation protein mass spectrometry analysis was performed according to according to the manufacturer operation manual(Shanghai Majorbio Bio-pharm Technology Co.,Ltd).

**Single cell RNA-seq** Single-cell suspensions were loaded into 10×Genomics Chromium platform, and sequencing libraries were constructed with reagents from a Chromium Single Cell 3′ Reagent Kit v3 (10× Genomics) according to the manufacturer’s instructions.

**ATAC-Seq**  ATAC-seq libraries of cells were prepared as the manufacturer’s protocol (Cat. No. K1157, APExBIO). The nuclei were then incubated with the transposase enzyme and tagmentation buffer at 37 °C for 30 min. The fragmented DNA was then purified using MinElute Reaction Cleanup Kit (Cat No. 28204, QIAGEN) in a final volume of 10 μL. The DNA libraries were amplified for 10 cycles and sequenced using Illumina NovaSeq 6000 with paired-end 2×150 as the sequencing mode. The clusterProfiler was used to perform functional enrichment analysis for the annotated significant peaks, the potential peaks in identified modules based on gene ontology (GO) and KEGG pathway categories. HOMER(v4.11) was utilized to Motif prediction of peaks.

**Supplemental Figures and Figure Legends**

**
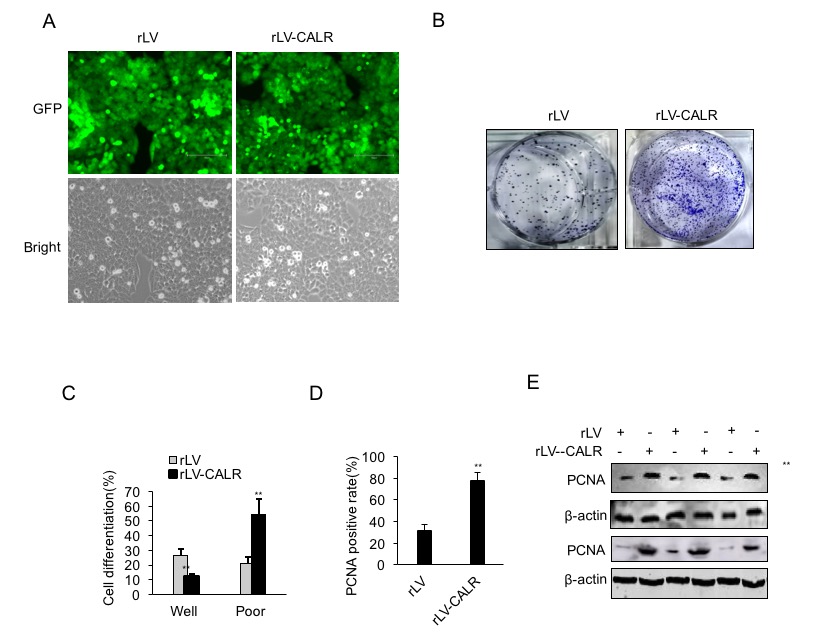
**

**Figure S1 Excessive** **CALR promotes the growth of liver cancer cells *in vitro and in vivo*.** A. Huh7 cells were infected with rLV, rLV-CALR and the pictures were taken under fluorescence microscope. B. The colony forming ability of cells was measured.. The photos of plate colonies. C. The transplanted tumor tissue sections (4 μ m) fixed in 4% formaldehyde and embedded in paraffin were stained with hematoxylin eosin (HE). D. anti-PCNA immunohistochemical staining.The values of each group were expressed as mean ± SD (n = 6), * *, P < 0.01, and *, P < 0.05.. E.Western blot with anti-PCNA.β-actin as internal control.


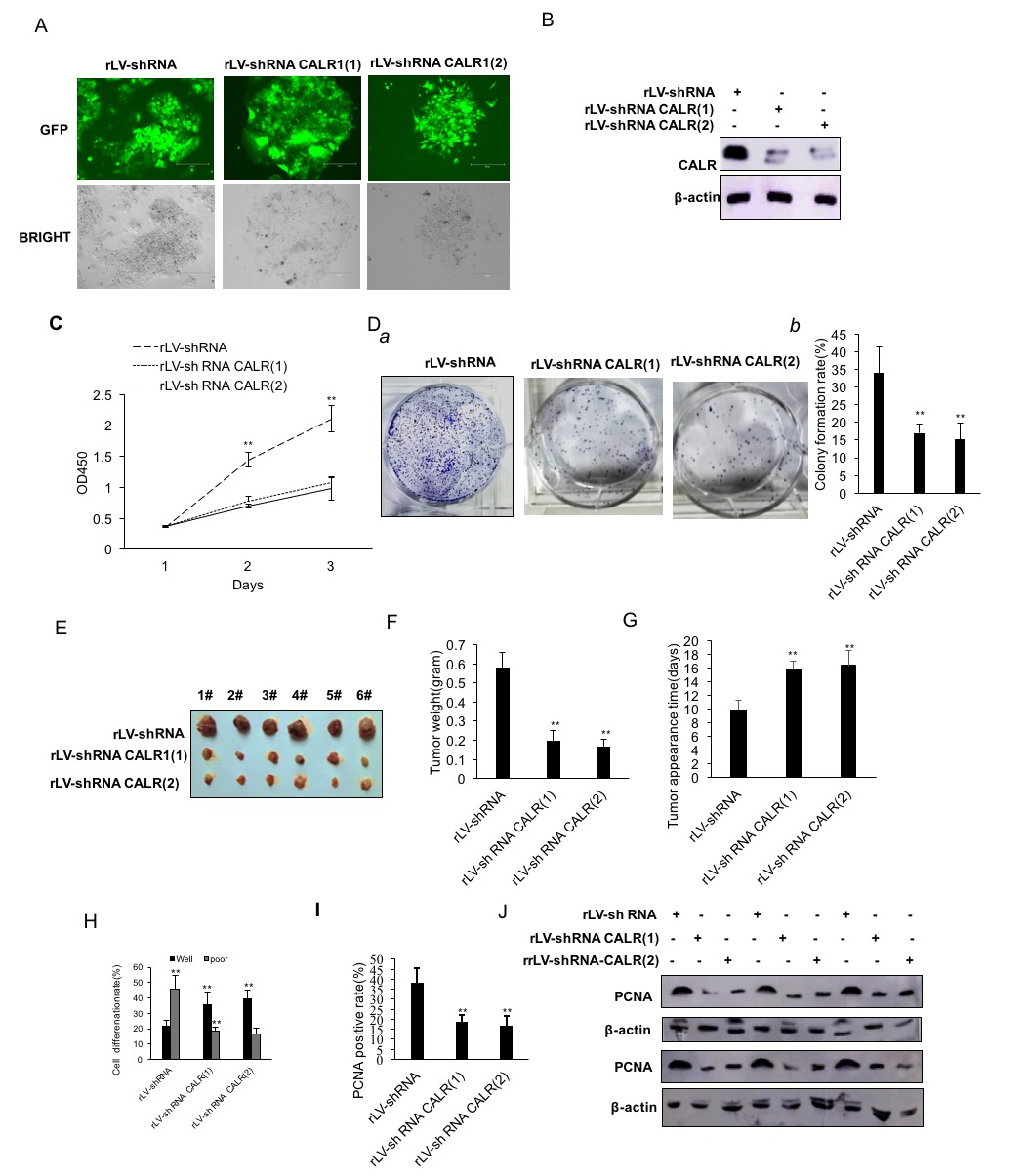


**FigureS2 CALR knockdown inhibits the growth of liver cancer cells *in vitro and in vivo*.** A. Huh7 cells were infected with rLV-shRNA, rLV-shRNA and the pictures were taken under fluorescence microscope. B. The CALR was detected by Western blot with anti-CALR. β-actin was used as internal reference gene.C. CCK8 method was used to determine the cell proliferation ability. The values of each group were expressed as mean ± SD (n =6), * *, P < 0.01, and *, P < 0.05. D. The colony forming ability of cells was measured. a. photos of plate colonies. b. analysis of colony forming ability of cells. The values of each group were expressed as mean ± standard deviation (bar ± SD, n = 6), * *, P < 0.01, *, P < 0.05. E. the xenograft tumor was dissected. F. Comparison of tumor size (g). G. The appearance time of tumor (days). The values of each group were expressed as mean ± SD (n = 6), * *, P < 0.01, and *, P < 0.05, respectively. H The transplanted tumor tissue sections (4 μ m) fixed in 4% formaldehyde and embedded in paraffin were stained with hematoxylin eosin (HE). The values of each group were expressed as mean ± SD (n = 6), * *, P < 0.01, and *, P < 0.05.. I. anti-PCNA immunohistochemical staining. The values of each group were expressed as mean ± SD (n = 6), * *, P < 0.01, and *, P < 0.05.. J.Western blot with anti-PCNA.β-actin as internal control.

**
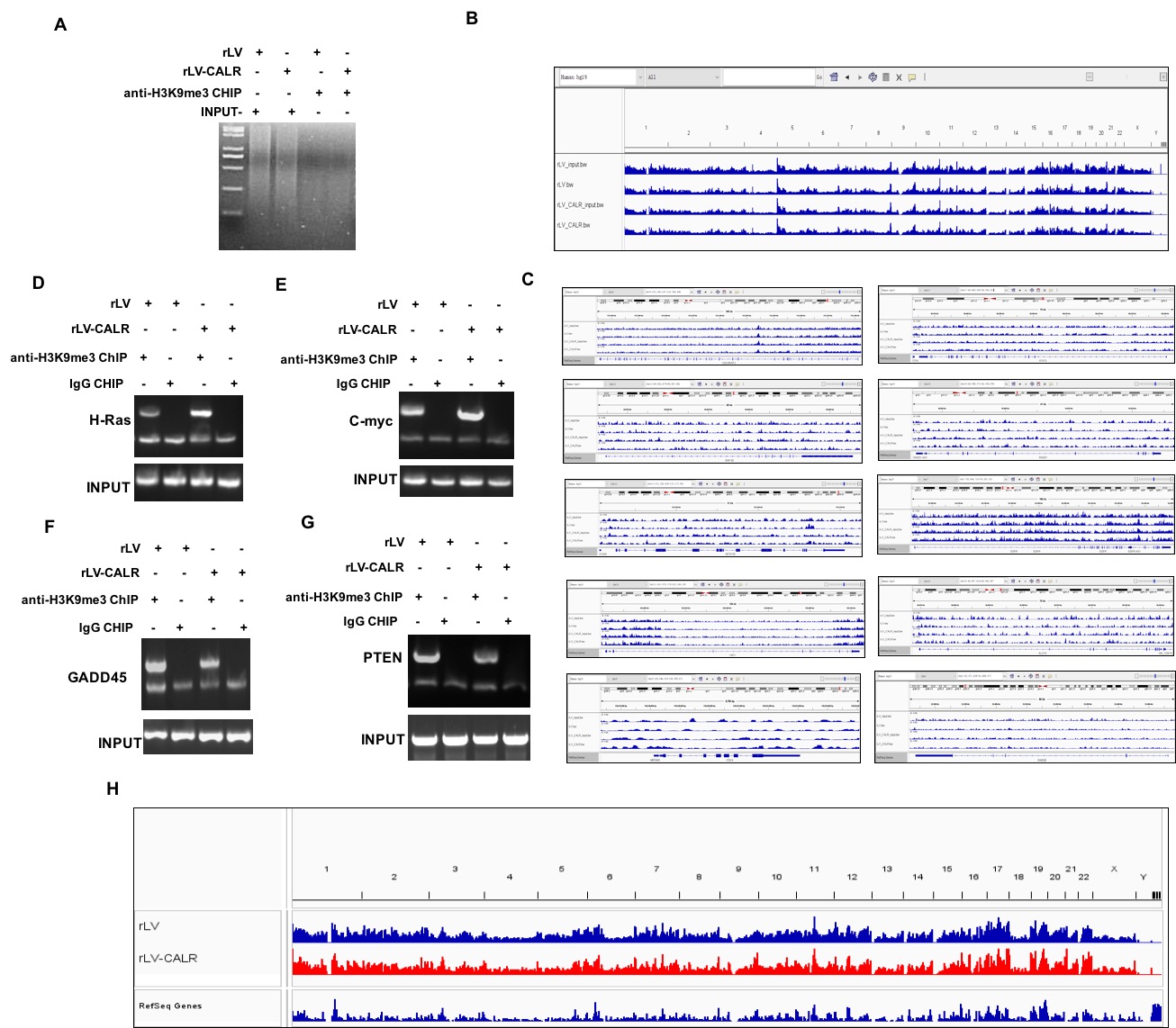
**

**FigureS3 Chromatin immunoprecipitation sequencing (ChIP-Seq) with anti-H3K9me3 high-throughput analysis was performed in human liver cancer cells.** A. Cells were cross-linked by formaldehyde of 1% and DNA was extracted. Then the DNA fragments after ultrasonic fragmentation were identified by 1% agarose gel electrophoresis. B. IGV browser interface (Demo): visualization of the reads of the modification distribution of H3K9me3 on 23 pairs of chromosomes in rLV group and rLV-CALR group.C. IGV browser interface (Demo): visualization of the reads of modification distribution of H3K9me3 on gene region. D. CHIP assay with anti-H-Ras. E. CHIP assay with anti-C-myc. G. CHIP assay with anti-GADD45. H. CHIP assay with anti-PTEN. I. ATAC-seq analysis.


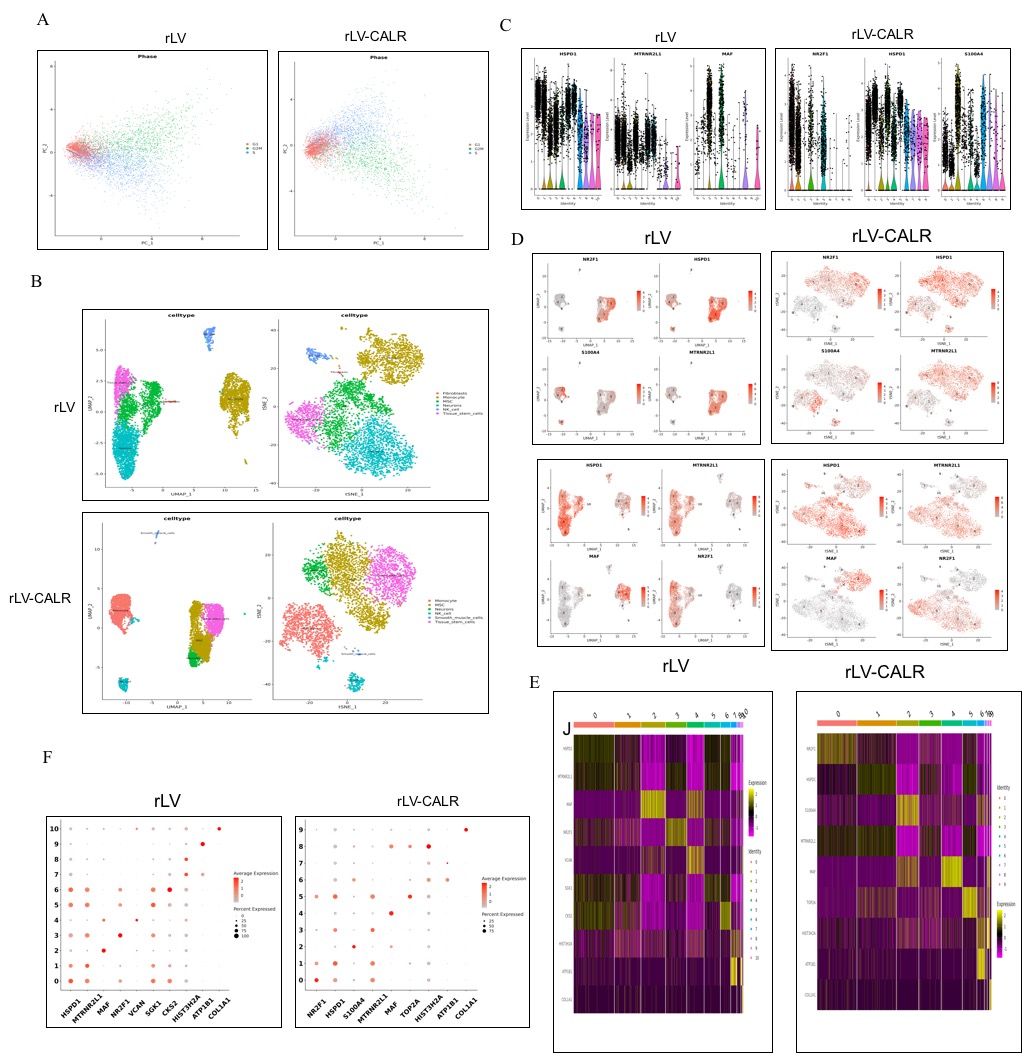


**Figure S4 CALR influences on liver cancer heterogeneity** A.Cell cycle PCA dimensionality reduction clustering. Red clusters represent cells in the G1 phase, green clusters represent cells in the G2M phase, and blue clusters represent cells in the S phase.B Dimensionality Reduction Clustering UMAP & tSNE Diagram.C The violin plot of the Cluster marker gene in cell clusters. Select the highly expressed genes in each cluster top (sorted by avg.log2FC) and observe their expression in different clusters.D The tSNE/UMAP graph of cell clusters marker genes. The top (sorted by avg_log2FC) highly expressed genes for each cluster are specifically marked, which can provide the expression patterns of each differential gene in each cluster. E. the heatmap of cell clusters marker genes for the expression levels of different genes. The horizontal axis represents different color blocks representing different clusters, and each line represents a cell. The vertical axis is the gene name. F.The dotplot graph of cell clusters marker genes for the expression levels of different genes. The horizontal axis is the gene name, and the vertical axis is the cluster number. The dots in the graph represent the expression levels of each gene in each cluster.


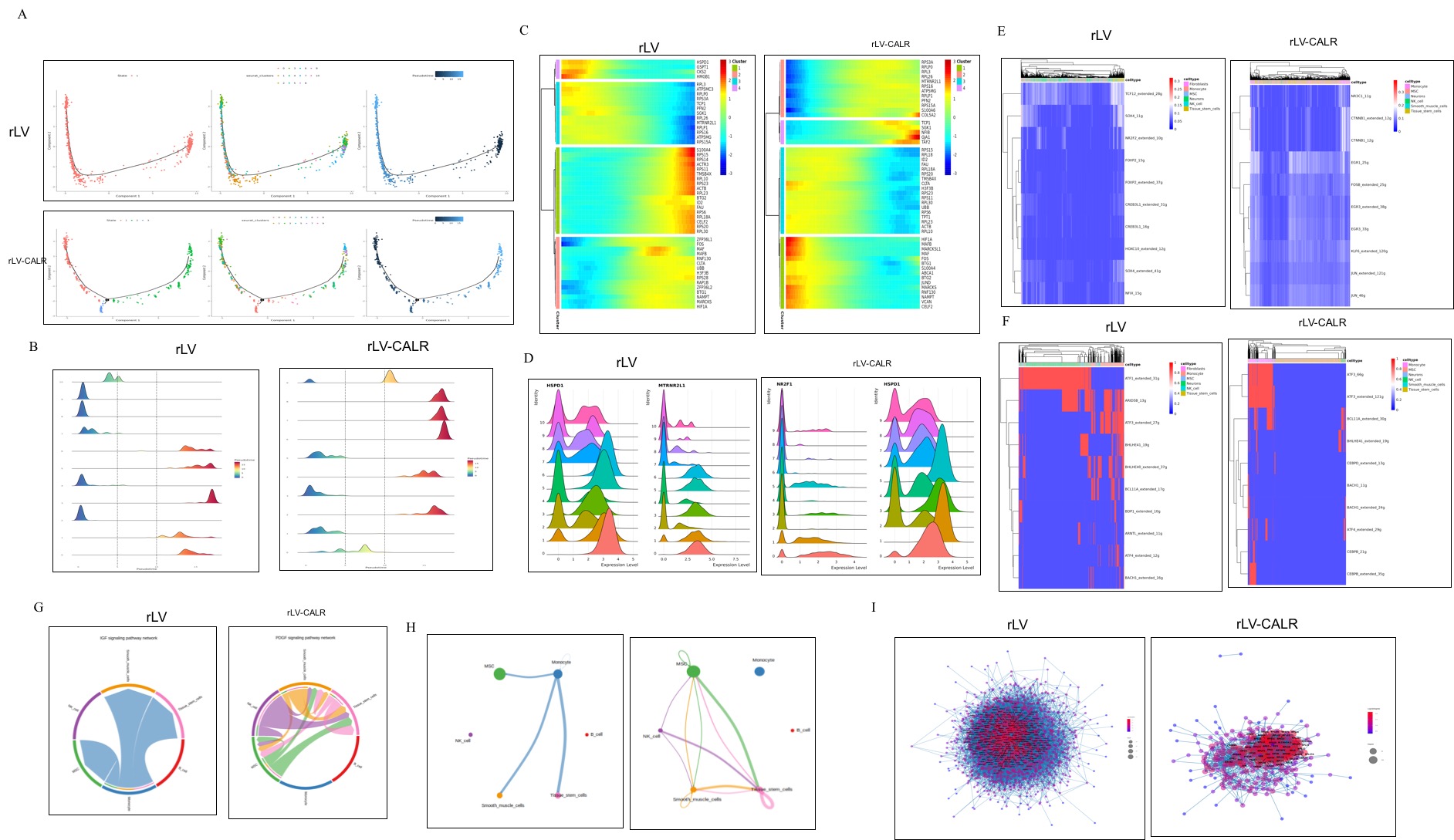


**Figure S5 CALR impacts on the liver cancer microenvironment.** A The trajectory plot of cluster using Monocle2 software. The trajectory lines of the cells in each cluster are marked with numbers. B. The ridge plot of simulated time trajectories. Different colors represent different clusters. The horizontal axis represents the cluster name, and the vertical axis represents the simulated time trajectory.C.The differential gene heatmap of simulated time trajectories. The horizontal axis is the time sequence, and the right vertical axis is the gene name, The size of the dots in the figure represents the number of genes, and the color ranges from red to blue to represent the degree of enrichment significance from high to low. D. Ridge diagram of genes with differential differentiation fate. The horizontal axis represents the time series, the right side of the vertical axis represents gene names, and the left color block represents different cell clusters.E-F.The heat map of partial transcription factor regulation analysis .different cell types on the horizontal axis and transcription factors on the vertical axis. The heatmap from blue to red represents the increase in cell activity from weak to strong.G. Receptor Circle Diagram.H.Receptor Loop Diagram..I Protein interaction network diagram.


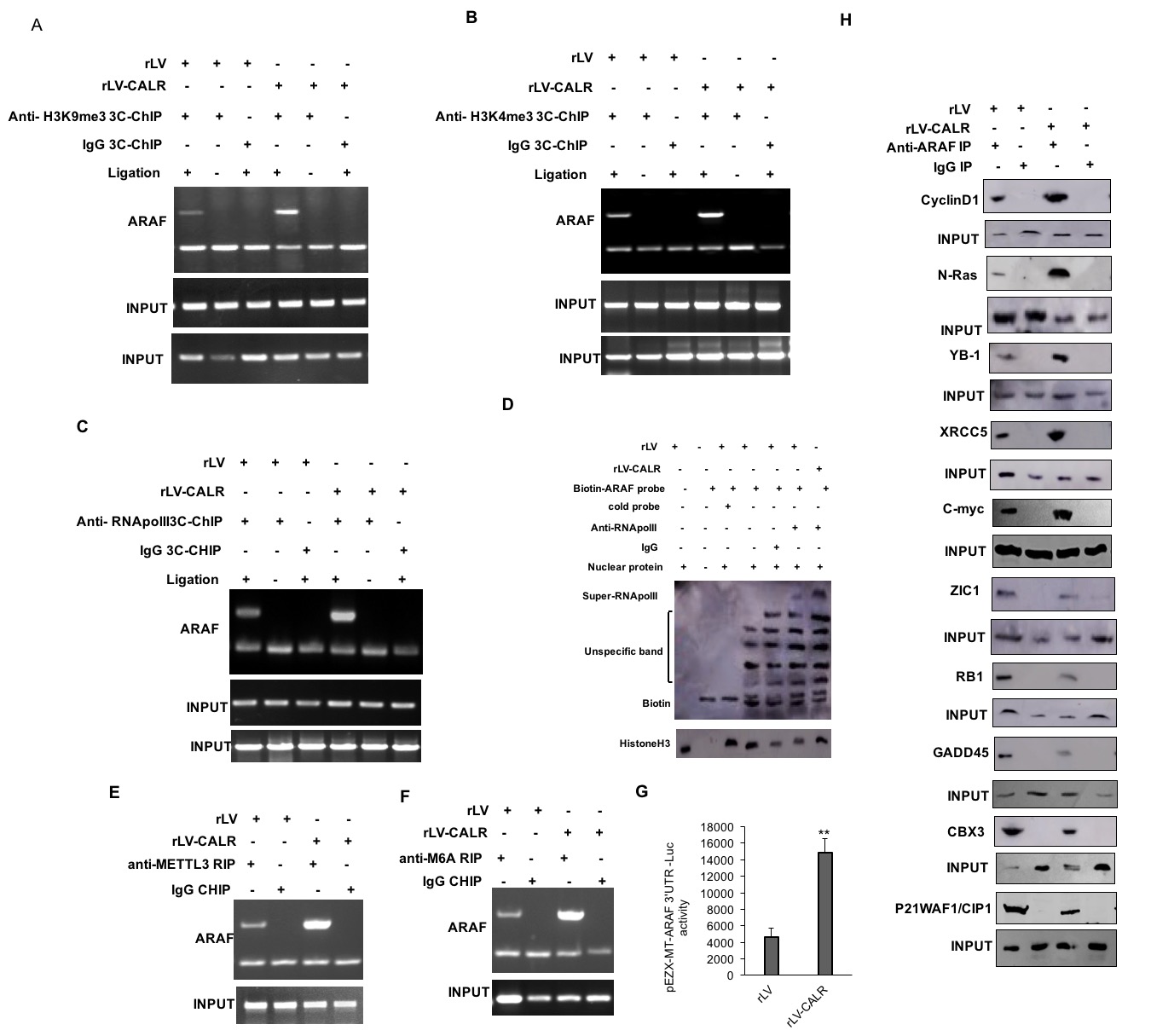


**FigureS6 CALR enhances the expression of ARAF in liver cancer.** A-C.. 3C-Chromatin immunocoprecipitation (CHIP) analysis was performed by anti-H3K9me3, anti-H3K4me3, anti-RNApolII, respectively. The PCR amplification was carried out by using primers designed according to the DNA of ARAF promoter and enhancer region. IgG 3C-CHIP was used as the negative control. D.Super-DNA-EMSA assay with anti-RNA polII and ARAF promote probe. E.RIP assay with anti-METTL3. F.RIP assay with anti-M6A.G. The assay of pEZX-MT-ARAF-3’UTR-Luc activity. H. Protein Co-immunocoprecipitation (Co-IP) analysis was performed by anti-ARAF. IgG Co-IP was used as the negative control.


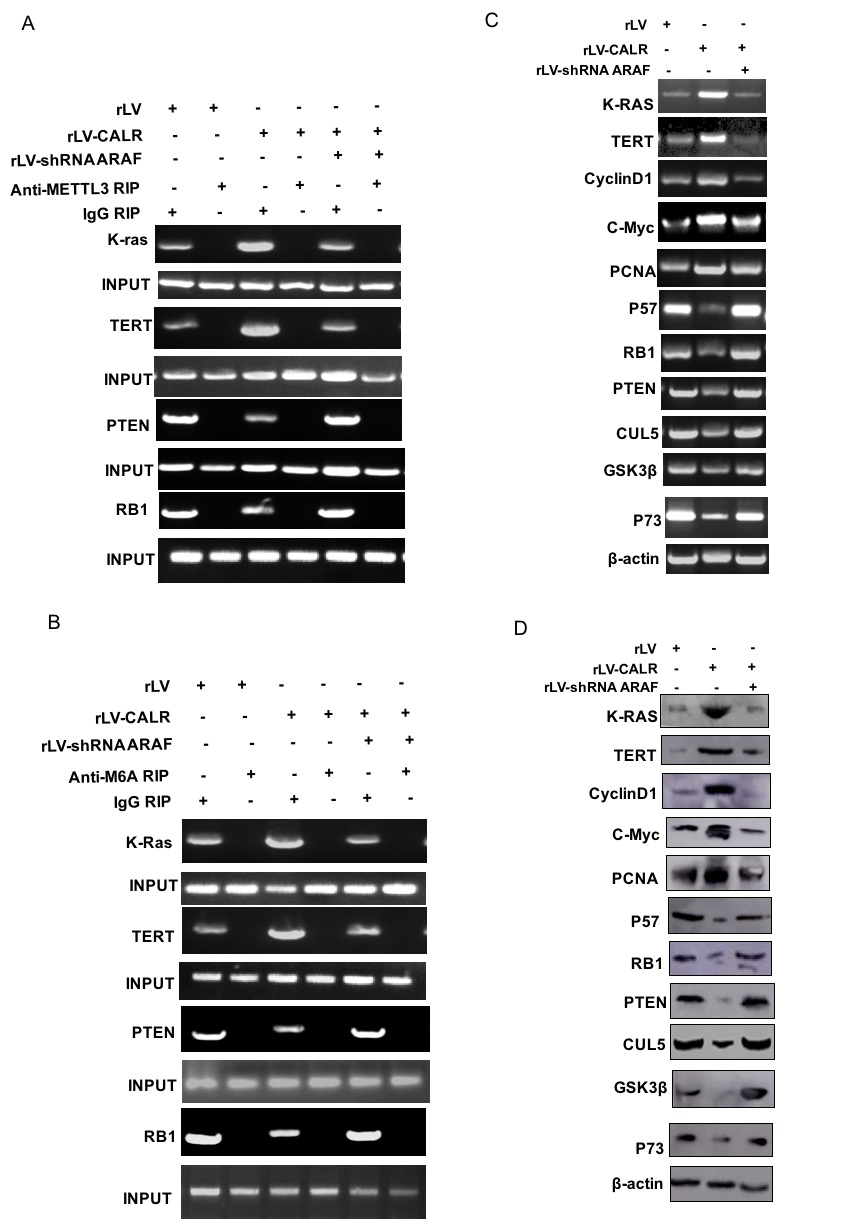


**FigureS7. CALR alters gene expression dependent on ARAF . A**. RIP assay with anti-METTL3. B. RIP assay with anti-M6A. C.The transcriptional ability of genes were detected by RT-PCR. β-actin was used as the internal reference gene. D. The translational ability of genes were detected by Western blot. β-actin was used as the internal reference gene.


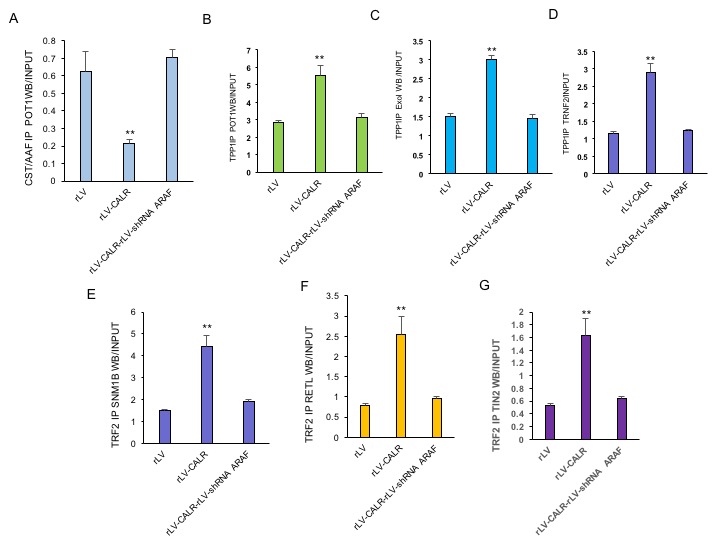


**Fig.S8.** Protein Co-immunocoprecipitation (Co-IP) analysis was performed. IgG Co-IP was used as the negative control. b. Gray scan analysis of positive bands. Each experiment was repeated three times. The values of each group were expressed as mean ± SD (n=3), * *, P < 0.01, *, P < 0.05.


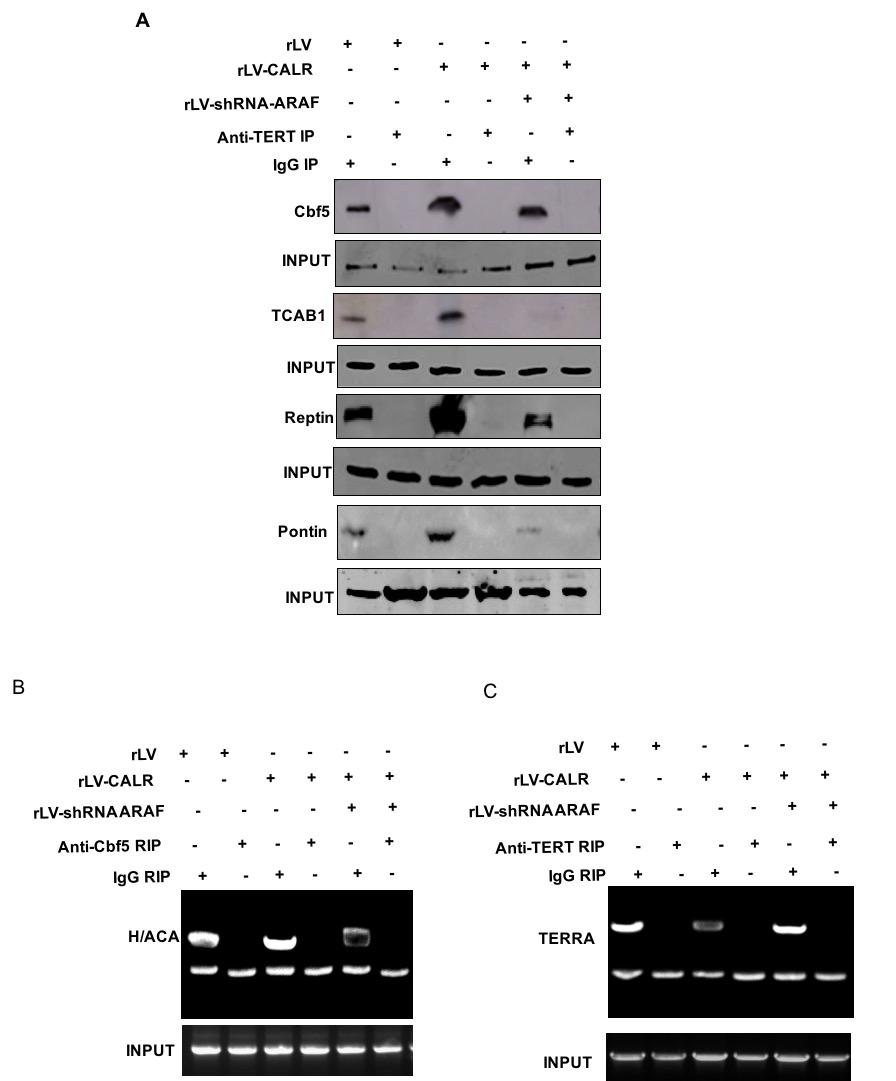


**FigureS10. CALR activates telomere via ARAF .** A. Protein Co-immunocoprecipitation (Co-IP) analysis was performed. IgG Co-IP was used as the negative control. B..RNA immunocoprecipitation (RIP) analysis was performed by anti-TERT. The RT-PCR amplification was carried out by using primers designed according to TERRA cDNA. IgG RIP was used as the negative control. C.RNA immunocoprecipitation (RIP) analysis was performed by anti-Cbf5. The RT-PCR amplification was carried out by using primers designed according to H/ACA cDNA. IgG RIP was used as the negative control.


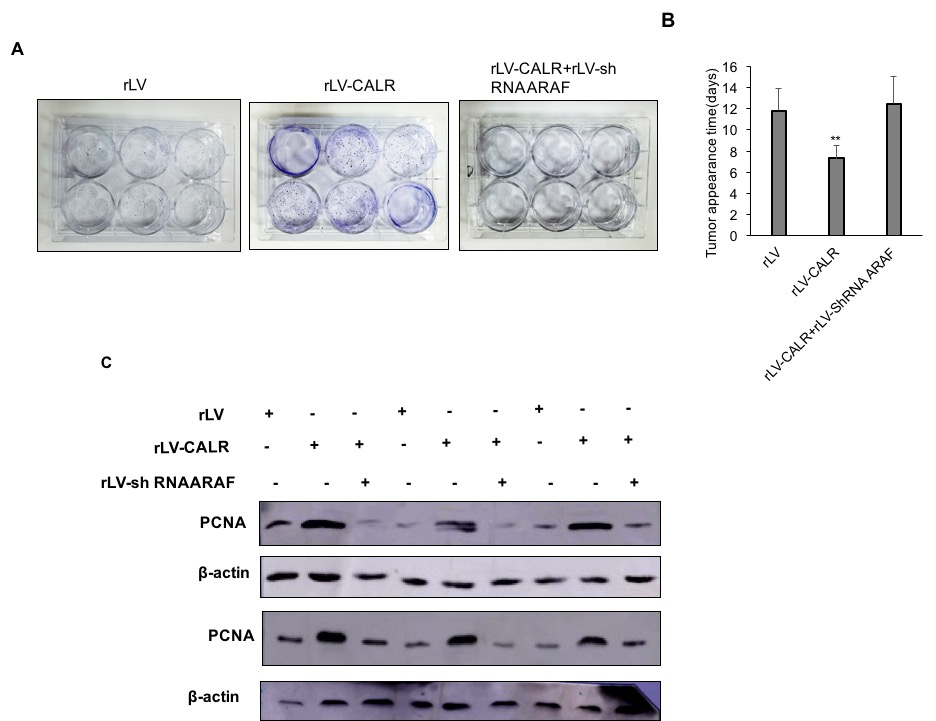


**Figure9. ARAF determines the cancerous functions of CALR** A..cellular colony pictures. B. Comparison of xenograft tumor appearance time(days).C. Western blot with anti-PCNA. β-actin was used as the internal reference gene.

**Discussion**

To date, we clearly demonstrate CALR accelerates the growth of liver cancer cells by enhancing ARAF expression and telomere functions**.** The first discovery provides a basis for the prevention and treatment of human liver cancer.

Calreticulin (CALR) is an endoplasmic reticulum (ER) protein involved in a range of cellular processes. In healthy cells, CALR functions as a chaperone and Ca2+ buffer, aiding in the proper folding and assembly of glycoproteins within the ER**[1]**. Pecquet et al. reported that hematopoietic cells with CALR mutations can secrete a soluble protein **[2]**. CALR can be translocated from the endoplasmic reticulum to the cell surface through co-localization with PDIA3**[3]** and supports anti-tumor immune reactions**[4]**. CALR signature from the antigen presentation machinery as a predictor of immunotherapy response**[5]**.Kristin et al. reported that CALR mutations affected the spindle assembly checkpoint, leading to erroneous mitosis**[6]**. Sun et al. found that TMCO1, by interacting with CALR to influence VDAC1, plays a crucial role in ovarian cancer progression **[7]**. Loss-of-function CALR mutations not only impair cellular homeostasis but also compromise natural **[8，9]**. Calreticulin (CALR) frameshift mutations are a recurrent driver in myeloproliferative neoplasms (MPNs) **[10-12]**. CALR drivives the development of myeloproliferative neoplasms**[13]**. he CALR-MPL complex forms in the endoplasmic reticulum, triggering continuous signaling **[14]**. In this study, we demonstrate that CALR accelerates the growth of liver cancer cells by enhancing expression of ARAF. These results provide basis for research on liver cancer prevention and treatment.

Obviously, our findings are noteworthy that CALR effect on epigenetic regulation in human liver cancer cells. For examples, CALR affects the modification of H3K9me3 on some genes promoter region, Importantly, our observations suggest that CALR affects transcriptome and proteome in liver cancer. Moreover, CALR regulates some signaling pathways network**.** Ultimately, CALR accelerates the growth ability of liver cancer cells to grow *in vivo and in vitro.* CALR mutants also exhibit oncogenic potential through homodimerization, epigenetic regulation changes, and defective calcium storage. The World Health Organization (WHO) currently recognizes CALR mutations as a critical diagnostic marker for determining myeloproliferative neoplasms **[15]**. CALR facilitates BC progression by promoting the BCSC phenotype through Wnt/β-catenin signaling [**16,17]**. miR-1307 facilitates the development of hepatocellular carcinoma through the CALR-OSTC-endoplasmic reticulum protein folding pathway**[18]**. S100A8 leads to abnormal characteristics in myeloproliferative neoplasms (MPNs) with CALR mutations**[19]**. CALR mutations are linked to increased expression of Retinoid X Receptor Alpha **[20]**. CALR influences NKTCL cell growth through G1-to-S cell cycle regulation **[21]**. CALR attracts and activates microglia while also inhibiting amyloid-beta aggregation **[22]**.The nuclear translocation of CALR reduces the CaM/CaMKII/CREB signaling pathway, improving chemosensitivity in hepatocellular carcinoma cells **[23,24]**. Our results are consistent with these reports, which needs further study.

Strikingly, our results suggest that CALR enhances the expression of ARAF and the in liver cancer. The binding ability of H3K9me3, H3K4me3, RNApolII to the promoter region of ARAF , and the methylation modification ability of ARAF mRNA were significantly increased in rLV-CALR group compared with rLV group. The expression of ARAF was significantly increased in rLV-CALR group compared with rLV group. Furthermore, the interaction between ARAF and CyclinD1, N-Ras, YB-1, XRCC5, C-myc were significantly increased，It is worth mentioning that CALR enhances autophagy through ARAF. ARAF protein kinase activates RAS by opposing its interaction with the RASGAP protein, NF1. **[25]**. ARAF inhibited neuregulin 1 (hNRG1)-mediated AKT activation through controlling ERBB3 expression **[26]**. ARAF plays a role in promoting GBC cell growth and metastasis**[27]**.The dimerization of the kinase ARAF facilitates the activation of the MAPK pathway [**28**].Linc01232 facilitates the metastasis of pancreatic cancer via ARAF activation**[29]**. ARAF governs trophoblast migration by EIF5A1**[30]**.NONO was found to interact with and stabilize both CRAF and ARAF in melanoma cells **[31]**. Our results are consistent with these reports, which needs further study.

*In conclusions,* CALR accelerates the growth of liver cancer cells by enhancing ARAF and autophagy. These results provide basis for research on liver cancer prevention and treatment.

**Reference**

1.M. Michalak, E. F. Corbett, N. Mesaeli, K. Nakamura and M. Opas. Calreticulin: one protein, one gene, many functions. The Biochemical Journal, 1999, 344 Pt 2(Pt 2): 281-292.

2. C. Pecquet, N. Papadopoulos, T. Balligand, I. Chachoua, A. Tisserand, G. Vertenoeil, A. Nédélec, D. Vertommen, A. Roy, C. Marty, H. Nivarthi, J. P. Defour, M. El-Khoury, E. Hug, A. Majoros, E. Xu, O. Zagrijtschuk, T. E. Fertig, D. S. Marta, H. Gisslinger, B. Gisslinger, M. Schalling, I. Casetti, E. Rumi, D. Pietra, C. Cavalloni, L. Arcaini, M. Cazzola, N. Komatsu, Y. Kihara, Y. Sunami, Y. Edahiro, M. Araki, R. Lesyk, V. Buxhofer-Ausch, S. Heibl, F. Pasquier, V. Havelange, I. Plo, W. Vainchenker, R. Kralovics and S. N. Constantinescu. Secreted mutant calreticulins as rogue cytokines in myeloproliferative neoplasms. Blood, 2023, 141(8): 917-929.

3. T. Panaretakis, N. Joza, N. Modjtahedi, A. Tesniere, I. Vitale, M. Durchschlag, G. M. Fimia, O. Kepp, M. Piacentini, K. U. Froehlich, P. van Endert, L. Zitvogel, F. Madeo and G. Kroemer. The co-translocation of ERp57 and calreticulin determines the immunogenicity of cell death. Cell Death and Differentiation, 2008, 15(9): 1499-1509.

4. K. Wang, H. Li, R. Chen, Y. Zhang, X.-X. Sun, W. Huang, H. Bian and Z.-N. Chen. Combination of CALR and PDIA3 is a potential prognostic biomarker for non-small cell lung cancer. Oncotarget, 2017, 8(57): 96945-96957.

5. R. Chen, H. Zhang, W. Wu, S. Li, Z. Wang, Z. Dai, Z. Liu, J. Zhang, P. Luo, Z. Xia and Q. Cheng. Antigen Presentation Machinery Signature-Derived CALR Mediates Migration, Polarization of Macrophages in Glioma and Predicts Immunotherapy Response. Frontiers In Immunology, 2022, 13: 833792.

6.K. Holl, N. Chatain, S. Krapp, J. Baumeister, T. Maié, S. Schmitz, A. Scheufen, N. Brock, S. Koschmieder and D. Moreno-Andrés. Calreticulin and JAK2V617F driver mutations induce distinct mitotic defects in myeloproliferative neoplasms. Sci Rep, 2024, 14(1): 2810.

7.G. Sun, S. Gong, S. Lan, Y. He, Y. Sun and Z. Zhang. TMCO1 regulates cell proliferation, metastasis and EMT signaling through CALR, promoting ovarian cancer progression and cisplatin resistance. Cell Mol Biol (Noisy-le-grand), 2024, 70(1): 99-109.

8.J. Fucikova, R. Spisek, G. Kroemer and L. Galluzzi. Calreticulin and cancer. Cell Res, 2021, 31(1): 5-16.

9. M. O. Holmström, M. Andersen, S. Traynor, S. M. Ahmad, T. L. Lisle, J. Handlos Grauslund, V. Skov, L. Kjær, J. T. Ottesen, M. F. Gjerstorff, H. C. Hasselbalch and M. H. Andersen. Therapeutic cancer vaccination against mutant calreticulin in myeloproliferative neoplasms induces expansion of specific T cells in the periphery but specific T cells fail to enrich in the bone marrow. Frontiers In Immunology, 2023, 14: 1240678.

10. K. Minakawa, T. Yokokawa, K. Ueda, O. Nakajima, T. Misaka, Y. Kimishima, K. Wada, Y. Tomita, S. Miura, Y. Sato, K. Mimura, K. Sugimoto, K. Nakazato, K. E. Nollet, K. Ogawa, T. Ikezoe, Y. Hashimoto, Y. Takeishi and K. Ikeda. Myeloproliferative neoplasm-driving Calr frameshift promotes the development of pulmonary hypertension in mice. Journal of Hematology & Oncology, 2021, 14(1): 52.

11. N. Papadopoulos, A. Nédélec, A. Derenne, T. A. Şulea, C. Pecquet, I. Chachoua, G. Vertenoeil, T. Tilmant, A.-J. Petrescu, G. Mazzucchelli, B. I. Iorga, D. Vertommen and S. N. Constantinescu. Oncogenic CALR mutant C-terminus mediates dual binding to the thrombopoietin receptor triggering complex dimerization and activation. Nature Communications, 2023, 14(1): 1881.

12.K. Olschok, L. Han, M. A. S. de Toledo, J. Böhnke, M. Graßhoff, I. G. Costa, A. Theocharides, A. Maurer, H. M. Schüler, E. M. Buhl, K. Pannen, J. Baumeister, M. Kalmer, S. Gupta, P. Boor, D. Gezer, T. H. Brümmendorf, M. Zenke, N. Chatain and S. Koschmieder. CALR frameshift mutations in MPN patient-derived iPSCs accelerate maturation of megakaryocytes. Stem Cell Reports, 2021, 16(11): 2768-2783.

13.J. Ibarra, Y. A. Elbanna, K. Kurylowicz, M. Ciboddo, H. S. Greenbaum, N. S. Arellano, D. Rodriguez, M. Evers, A. Bock-Hughes, C. Liu, Q. Smith, J. Lutze, J. Baumeister, M. Kalmer, K. Olschok, B. Nicholson, D. Silva, L. Maxwell, J. Dowgielewicz, E. Rumi, D. Pietra, I. C. Casetti, S. Catricala, S. Koschmieder, S. Gurbuxani, R. K. Schneider, S. A. Oakes and S. E. Elf. Type I but Not Type II Calreticulin Mutations Activate the IRE1α/XBP1 Pathway of the Unfolded Protein Response to Drive Myeloproliferative Neoplasms. Blood Cancer Discovery, 2022, 3(4): 298-315.

14.Y. Edahiro, M. Araki and N. Komatsu. Mechanism underlying the development of myeloproliferative neoplasms through mutant calreticulin. Cancer Science, 2020, 111(8): 2682-2688.

15.T. Belčič Mikič, T. Pajič, S. Zver and M. Sever. The Contemporary Approach to CALR-Positive Myeloproliferative Neoplasms. Int J Mol Sci, 2021, 22(7).

16. X. Liu, P. Xie, N. Hao, M. Zhang, Y. Liu, P. Liu, G. L. Semenza, J. He and H. Zhang. HIF-1-regulated expression of calreticulin promotes breast tumorigenesis and progression through Wnt/β-catenin pathway activation. Proceedings of the National Academy of Sciences of the United States of America, 2021, 118(44).

17.D. Ivanov, J. D. Milosevic Feenstra, I. Sadovnik, H. Herrmann, B. Peter, M. Willmann, G. Greiner, K. Slavnitsch, E. Hadzijusufovic, T. Rülicke, M. Dahlhoff, G. Hoermann, S. Machherndl-Spandl, G. Eisenwort, M. Fillitz, T. Sliwa, M.-T. Krauth, P. Bettelheim, W. R. Sperr, E. Koller, M. Pfeilstöcker, H. Gisslinger, F. Keil, R. Kralovics and P. Valent. Phenotypic characterization of disease-initiating stem cells in JAK2- or CALR-mutated myeloproliferative neoplasms. American Journal of Hematology, 2023, 98(5): 770-783.

18.S. Xie, X. Jiang, R. Qin, S. Song, Y. Lu, L. Wang, Y. Chen and D. Lu. miR-1307 promotes hepatocarcinogenesis by CALR-OSTC-endoplasmic reticulum protein folding pathway. IScience, 2021, 24(11): 103271.

19. Y.-H. Wang, Y.-J. Chen, Y.-H. Lai, M.-C. Wang, Y.-Y. Chen, Y.-Y. Wu, Y.-R. Yang, H.-Y. Tsou, C.-P. Li, C.-C. Hsu, C.-E. Huang and C.-C. Chen. Mutation-Driven S100A8 Overexpression Confers Aberrant Phenotypes in Type 1 CALR-Mutated MPN. International Journal of Molecular Sciences, 2023, 24(10).

20. A. Guijarro-Hernández, C. Hurtado, M. J. Larráyoz, M. J. Calasanz and J. L. Vizmanos. CALR but Not JAK2 Mutations Are Associated with an Overexpression of Retinoid X Receptor Alpha in Essential Thrombocythemia. Cancers, 2024, 16(8).

21. Y. Zheng, C. Li, P. Xin, Q. Peng, W. Zhang, S. Liu and X. Zhu. Calreticulin increases growth and progression of natural killer/T-cell lymphoma. Aging, 2020, 12(23): 23822-23835.

22. K. M. Reid, E. J. A. Kitchener, C. A. Butler, T. O. J. Cockram and G. C. Brown. Brain Cells Release Calreticulin That Attracts and Activates Microglia, and Inhibits Amyloid Beta Aggregation and Neurotoxicity. Frontiers In Immunology, 2022, 13: 859686.

23. Y.-S. Liu, Y.-C. Chang, W.-W. Kuo, M.-C. Chen, T.-F. Wang, T.-S. Chen, Y.-M. Lin, C.-C. Li, P.-H. Liao and C.-Y. Huang. Calreticulin nuclear translocalization alleviates CaM/CaMKII/CREB signaling pathway to enhance chemosensitivity in HDAC inhibitor-resistant hepatocellular carcinoma cells. Aging, 2022, 14(12): 5097-5115.

24. Q. Ding, W. Tang, X. Li, Y. Ding, X. Chen, W. Cao, X. Wang, W. Mo, Z. Su, Q. Zhang and H. Guo. Mitochondrial-targeted brequinar liposome boosted mitochondrial-related ferroptosis for promoting checkpoint blockade immunotherapy in bladder cancer. Journal of Controlled Release : Official Journal of the Controlled Release Society, 2023, 363: 221-234.

25. W. Su, R. Mukherjee, R. Yaeger, J. Son, J. Xu, N. Na, N. Merna Timaul, J. Hechtman, V. Paroder, M. Lin, M. Mattar, J. Qiu, Q. Chang, H. Zhao, J. Zhang, M. Little, Y. Adachi, S.-W. Han, B. S. Taylor, H. Ebi, O. Abdel-Wahab, E. de Stanchina, C. M. Rudin, P. A. Jänne, F. McCormick, Z. Yao and N. Rosen. ARAF protein kinase activates RAS by antagonizing its binding to RASGAP NF1. Molecular Cell, 2022, 82(13).

26. J. Mooz, K. Riegel, H. Ps, A. Sadanandam, F. Marini, M. Klein, U. Werner, W. Roth, A. Wilken-Schmitz, I. Tegeder and K. Rajalingam. ARAF suppresses ERBB3 expression and metastasis in a subset of lung cancers. Science Advances, 2022, 8(11): eabk1538.

27. W. Lin, C. Tong, W. Zhang, W. Cen, Y. Wang, J. Li, Z. Zhu, J. Yu and B. Lu. Silencing ARAF Suppresses the Malignant Phenotypes of Gallbladder Cancer Cells. BioMed Research International, 2020, 2020: 3235786.

28. J. Mooz, T. K. Oberoi-Khanuja, G. S. Harms, W. Wang, B. S. Jaiswal, S. Seshagiri, R. Tikkanen and K. Rajalingam. Dimerization of the kinase ARAF promotes MAPK pathway activation and cell migration. Science Signaling, 2014, 7(337): ra73.

29. L.-D. Meng, G.-D. Shi, W.-L. Ge, X.-M. Huang, Q. Chen, H. Yuan, P.-F. Wu, Y.-C. Lu, P. Shen, Y.-H. Zhang, S.-J. Cao, Y. Miao, M. Tu and K.-R. Jiang. Linc01232 promotes the metastasis of pancreatic cancer by suppressing the ubiquitin-mediated degradation of HNRNPA2B1 and activating the A-Raf-induced MAPK/ERK signaling pathway. Cancer Letters, 2020, 494: 107-120.

30.[Jing Zhang](https://pubmed.ncbi.nlm.nih.gov/?term=Zhang+J&cauthor_id=30206208) ,Hui-Qin Mo,[Fu-Ju Tian](https://pubmed.ncbi.nlm.nih.gov/?term=Tian+FJ&cauthor_id=30206208), [Wei-Hong Zeng](https://pubmed.ncbi.nlm.nih.gov/?term=Zeng+WH&cauthor_id=30206208), [Xiao-Rui Liu](https://pubmed.ncbi.nlm.nih.gov/?term=Liu+XR&cauthor_id=30206208), [Xiao-Ling Ma](https://pubmed.ncbi.nlm.nih.gov/?term=Ma+XL&cauthor_id=30206208), [Xiao Li](https://pubmed.ncbi.nlm.nih.gov/?term=Li+X&cauthor_id=30206208), [Shi Qin](https://pubmed.ncbi.nlm.nih.gov/?term=Qin+S&cauthor_id=30206208) , [Cui-Fang Fan](https://pubmed.ncbi.nlm.nih.gov/?term=Fan+CF&cauthor_id=30206208), [Yi Lin](https://pubmed.ncbi.nlm.nih.gov/?term=Lin+Y&cauthor_id=30206208).EIF5A1 promotes trophoblast migration and invasion via ARAF-mediated activation of the integrin/ERK signaling pathway.Cell Death Dis.2018;9(9):926.

31.Feifei Zhang, [Xiaofeng Tang](https://pubmed.ncbi.nlm.nih.gov/?term=Tang+X&cauthor_id=34017080), [Song Fan](https://pubmed.ncbi.nlm.nih.gov/?term=Fan+S&cauthor_id=34017080) , [Xia Liu](https://pubmed.ncbi.nlm.nih.gov/?term=Liu+X&cauthor_id=34017080), [Jun Sun](https://pubmed.ncbi.nlm.nih.gov/?term=Sun+J&cauthor_id=34017080) , [Cheng Ju](https://pubmed.ncbi.nlm.nih.gov/?term=Ju+C&cauthor_id=34017080), [Yiping Liang](https://pubmed.ncbi.nlm.nih.gov/?term=Liang+Y&cauthor_id=34017080) , [Renfeng Liu](https://pubmed.ncbi.nlm.nih.gov/?term=Liu+R&cauthor_id=34017080), [Ruihao Zhou](https://pubmed.ncbi.nlm.nih.gov/?term=Zhou+R&cauthor_id=34017080) , [Bo Yu](https://pubmed.ncbi.nlm.nih.gov/?term=Yu+B&cauthor_id=34017080) , [Changhua Zhang](https://pubmed.ncbi.nlm.nih.gov/?term=Zhang+C&cauthor_id=34017080) , [Zhiping Zhang](https://pubmed.ncbi.nlm.nih.gov/?term=Zhang+Z&cauthor_id=34017080) , [Tiebang Kang](https://pubmed.ncbi.nlm.nih.gov/?term=Kang+T&cauthor_id=34017080) , [Guofu Huang](https://pubmed.ncbi.nlm.nih.gov/?term=Huang+G&cauthor_id=34017080) , [Xiao-Bin Lv](https://pubmed.ncbi.nlm.nih.gov/?term=Lv+XB&cauthor_id=34017080) .Targeting the p300/NONO axis sensitizes melanoma cells to BRAF inhibitors Oncogene .2021;40(24):4137-4150.
